# Combining radiomics with thyroid imaging reporting and data system to predict lateral cervical lymph node metastases in medullary thyroid cancer

**DOI:** 10.1186/s12880-024-01222-7

**Published:** 2024-03-18

**Authors:** Zhiqiang Liu, Xiwei Zhang, Xiaohui Zhao, Qianqian Guo, Zhengjiang Li, Minghui Wei, Lijuan Niu, Changming An

**Affiliations:** 1 Department of Radiation Oncology, National Cancer Center/National Clinical Research Center for Cancer/Cancer Hospital, Chinese Academy of Medical Sciences and Peking Union Medical College, Beijing, P.R. China; 2 Department of Head and Neck Surgical Oncology, National Cancer Center/National Clinical Research Center for Cancer/Cancer Hospital, Chinese Academy of Medical Sciences and Peking Union Medical College, No. 17, Panjiayuan Nanli, Chaoyang District, Beijing, 100021 P.R. China; 3 Department of Ultrasound, National Cancer Center/National Clinical Research Center for Cancer/Cancer Hospital, Chinese Academy of Medical Sciences and Peking Union Medical College, No. 17, Panjiayuan Nanli, Chaoyang District, Beijing, 100021 P.R. China; 4 Department of Head and Neck Surgical Oncology, National Cancer Center/National Clinical Research Center for Cancer/Cancer Hospital & Shenzhen Hospital, Chinese Academy of Medical Sciences and Peking Union Medical College, Beijing, P.R. China; 5 Department of Ultrasound, Qilu Hospital of Shandong University, Jinan, Shandong P.R. China

**Keywords:** Artificial intelligence, Radiomics, Medullary thyroid carcinoma, Ultrasound, Lateral cervical lymph node metastases

## Abstract

**Background:**

Medullary Thyroid Carcinoma (MTC) is a rare type of thyroid cancer. Accurate prediction of lateral cervical lymph node metastases (LCLNM) in MTC patients can help guide surgical decisions and ensure that patients receive the most appropriate and effective surgery. To our knowledge, no studies have been published that use radiomics analysis to forecast LCLNM in MTC patients. The purpose of this study is to develop a radiomics combined with thyroid imaging reporting and data system (TI-RADS) model that can use preoperative thyroid ultrasound images to noninvasively predict the LCLNM status of MTC.

**Methods:**

We retrospectively included 218 MTC patients who were confirmed from postoperative pathology as LCLNM negative (*n*=111) and positive (*n*=107). Ultrasound features were selected using the Student’s t-test, while radiomics features are first extracted from preoperative thyroid ultrasound images, and then a two-step feature selection approach was used to select features. These features are then used to establish three regularized logistic regression models, namely the TI-RADS model (TM), the radiomics model (RM), and the radiomics-TI-RADS model (RTM), in 5-fold cross-validation to determine the likelihood of the LCLNM. The Delong’s test and decision curve analysis (DCA) were used to evaluate and compare the performance of the models.

**Results:**

The ultrasound features of margin and TI-RADS level, and a total of 12 selected radiomics features, were significantly different between the LCLNM negative and positive groups (*p*<0.05). The TM, RM, and RTM yielded an averaged AUC of 0.68±0.05, 0.78±0.06, and 0.82±0.05 in the 5-fold cross-validation dataset, respectively. RM and RTM are statistically better than TM (*p*<0.05 and *p*<0.001) according to Delong test. DCA demonstrates that RTM brings more benefit than TM and RM.

**Conclusions:**

We have developed a joint radiomics-based model for noninvasive prediction of the LCLNM in MTC patients solely using preoperative thyroid ultrasound imaging. It has the potential to be used as a complementary tool to help guide treatment decisions for this rare form of thyroid cancer.

**Supplementary Information:**

The online version contains supplementary material available at 10.1186/s12880-024-01222-7.

## Background

Medullary Thyroid Carcinoma (MTC) is a rare type of thyroid cancer [1], accounting for 1%-2% of all thyroid cancers [2]. It has a higher propensity for lateral cervical lymph node metastases, accounting for 70% [3] of cases, as compared to other types of thyroid cancer. The American Thyroid Association (ATA) guidelines recommend surgery as the first-line therapy for definitive cure in MTC patients [4]. The standard surgical therapy for MTC typically includes total thyroidectomy and lymphadenectomy. However, the extent of cervical lymph node dissection is still a matter of debate, particularly regarding lateral cervical lymph node dissection [5–7]. The surgery decision making can affect the prognosis of MTC patients. Therefore, it is particularly important for preoperative assessment for lateral cervical lymph node metastases (LCLNM) in such patients.

Thyroid ultrasound is the first choice and a useful tool for diagnosing thyroid disease [6]. The Thyroid Imaging Reporting and Data System (TI-RADS) has been used as a standard method for the classification of thyroid nodules [8]. Due to its ease of use and clinical viability, TI-RADS has drawn considerable attention. The applicability of ultrasound-based TI-RADS in MTC patients has been evaluated and the relationship between ultrasound features and lymph node metastases has been assessed [9]. However, TI-RADS results are usually affected by the experience of reviewers in most cases, and information from ultrasound imaging has not been fully explored, at present only relying on the naked eye.

Radiomics can extract quantitative features from medical images that may reflect information about underlying pathophysiology that is not visible to the human eye [10]. In recent years, there have been numerous studies that highlight the emerging field of utilizing medical images with radiomics, combined with machine learning and deep learning to enhance the understanding and treatment of thyroid cancers, by providing personalized and detailed insights into tumor development [11–13]. Biomarkers based on quantitative radiomics and deep learning features from preoperative thyroid ultrasound have demonstrated promising outcomes for predicting distant metastases in follicular thyroid carcinoma [14], predicting thyroid malignancy [15–17], and predicting lymph nodes status of patients with papillary thyroid carcinoma [18–22].

Accurate prediction of LCLNM status in MTC patients can help guide surgical decisions and ensure that patients receive the most appropriate and effective surgery. To our knowledge, no studies have been published that use radiomics analysis to forecast LCLNM in MTC patients. The purpose of this study is to develop a separate biomarker which is radiomics-based for noninvasively predicting the LCLNM status of MTC using preoperative thyroid ultrasound images.

## Methods

### Patient selection and data acquisition

**Fig. 1:**
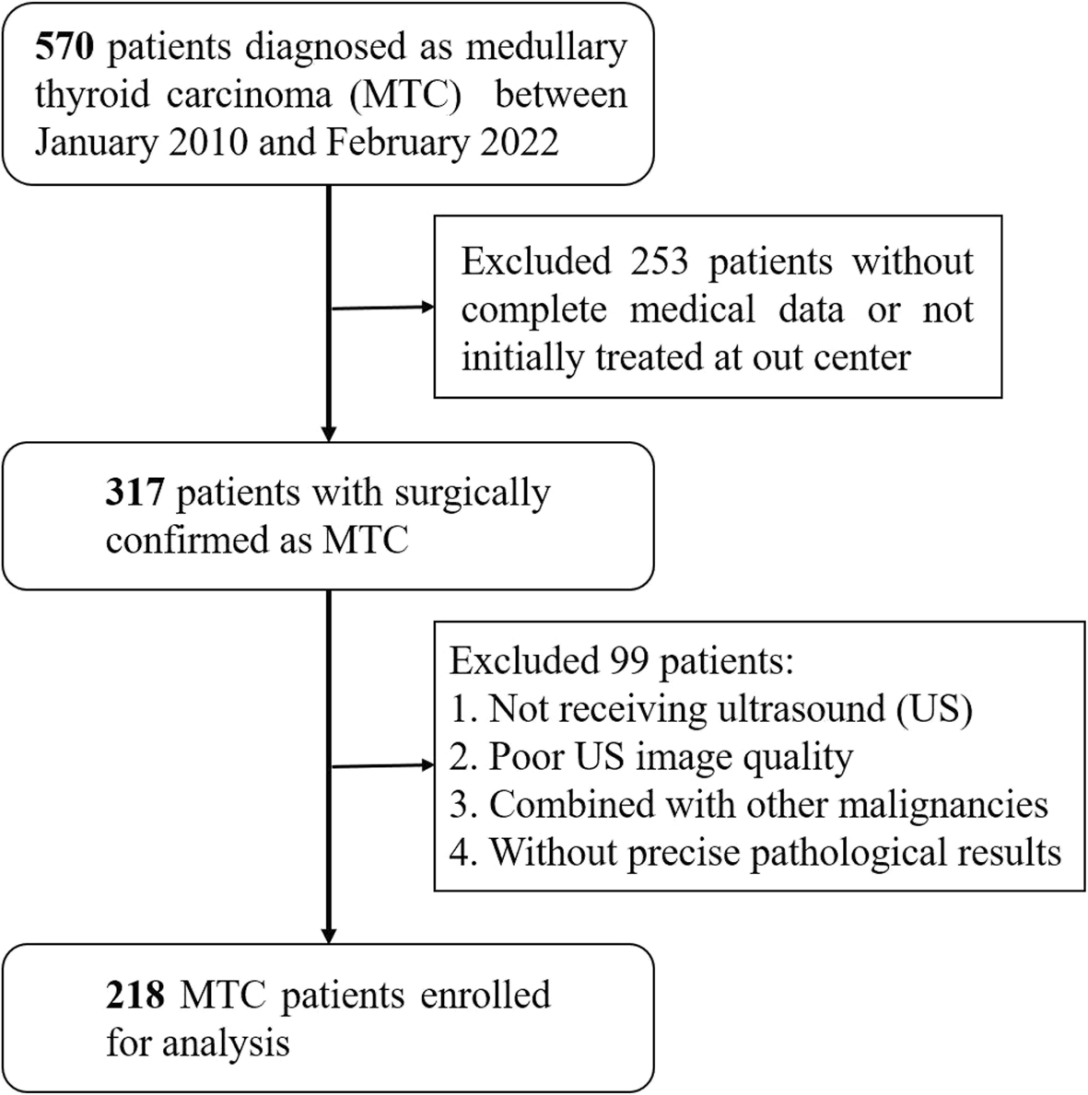
Flowchart of the study population

We retrospectively collected the patients with pathologically confirmed MTC between January 2010 and February 2022 at our medical center. Patients were included in this study if they: (1) received preoperative thyroid ultrasound with satisfactory image quality; (2) underwent initial surgical therapy in our medical center; (3) had complete medical records. A total of 218 eligible MTC patients were consecutively included and reviewed as shown in Fig. 1.

The age at diagnosis and sex, as well as the ultrasound features from American College of Radiology (ACR) TI-RADS and surgical pathology report were retrieved from electronic medical records. Table 1 shows an overview of patient characteristics. The LCLNM status (negative, N0/N1a or positive, N1b) were confirmed by surgical pathology.

**Table 1:** Patient characteristics of the 218 patients in medullary thyroid carcinoma (MTC) with lateral cervical lymph node metastases (LCLNM) negative (N0/N1a) and positive (N1b)

**Characteristics**	**LCLNM negative (*n*=111)**	**LCLNM positive (*n*=107)**	***p*-Value**
Age (years)^a^	49 (13~78)	50 (21~75)	0.995
Sex^b^			**<0.001**
Male	39 (35.1)	63 (58.9)	
Female	72 (64.9)	44 (41.1)	
**ACR TI-RADS**			
Composition^b^			0.222
Cystic or Spongiform	0 (0.0)	0 (0.0)	
Mixed cystic and nodule	11 (9.9)	5 (4.7)	
Solid	100 (90.1)	102 (95.3)	
Echogenicity^b^			0.491
Anechoic	0 (0.0)	0 (0.0)	
Hyperechoic or isoechoic	3 (2.7)	2 (1.9)	
Hypoechoic	89 (80.2)	80 (74.8)	
Very hypoechoic	19 (17.1)	25 (23.4)	
Shape^b^			0.392
Wider-than-tall	105 (94.6)	97 (90.7)	
Taller-than-wide	6 (5.4)	10 (9.3)	
Margin^b^			**<0.001**
Smooth or Ill-defined	22 (19.8)	7 (6.5)	
Lobulated or Irregular	87 (78.4)	69 (64.5)	
Extra-thyroidal extension	2 (1.8)	31 (29.0)	
Echogenic Foci^b^			0.393
None or Large comet-tail artifacts	49 (44.1)	38 (35.5)	
Macrocalcifications	41 (36.9)	48 (44.9)	
Peripheral(rim) calcifications	0 (0.0)	0 (0.0)	
Punctate echogenic foci	21 (18.9)	21 (19.6)	
TI-RADS Level^b^			**0.016**
TR1	0 (0.0)	0 (0.0)	
TR2	1 (0.9)	0 (0.0)	
TR3	5 (4.6)	0 (0.0)	
TR4	35 (31.5)	22 (20.6)	
TR5	70 (63.1)	85 (79.4)	

^a^Data are presented as medians with ranges in parentheses

^b^Data in parentheses are percentages

### Ultrasound images acquisition and preprocessing

One of four ultrasound scanners (GE logic 9-General Electric Company, USA; GE logic E9-General Electric Company, USA; Philips IU 22-Royal Dutch Philips Electronics Ltd, the Netherlands; and Siemens Acuson S2000-Siemens AG FWB:SIE, Germany) with a 5 to 12 MHz high-frequency linear transducer was used to screen thyroid pathologies. The patient was in a supine position while the thyroid gland was examined using a multi-section scan from the front of the neck. Ultrasound data (Table 1) included nodule composition, echogenicity, shape, margin, and echogenic foci. Two radiologists with 5 and 25 years of thyroid nodule diagnosis expertise blindly reviewed and recorded each patient's features. When two reviewers disagreed during valuation, a collaborative review was done and consensus values were used for statistical analysis.

A single ultrasound image that can represent the focus most comprehensively was selected for each tumor among all the images and then loaded into 3D Slicer software for manual segmentation. A region of interest (ROI) was manually delineated at the boundary of each primary tumor by one experienced radiologist. Then, the ultrasound images were cropped by using the ROI boundary box to remove useless information from the ultrasound images. Ultrasound images were normalized using the linear min-max normalization method:

1 $${I}_{normalized}=\frac{255\times ({I}_{original}-{\text{min}}\left({I}_{original}\right))}{{\text{max}}\left({I}_{original}\right)-{\text{min}}({I}_{original})}$$

**Fig. 2:**
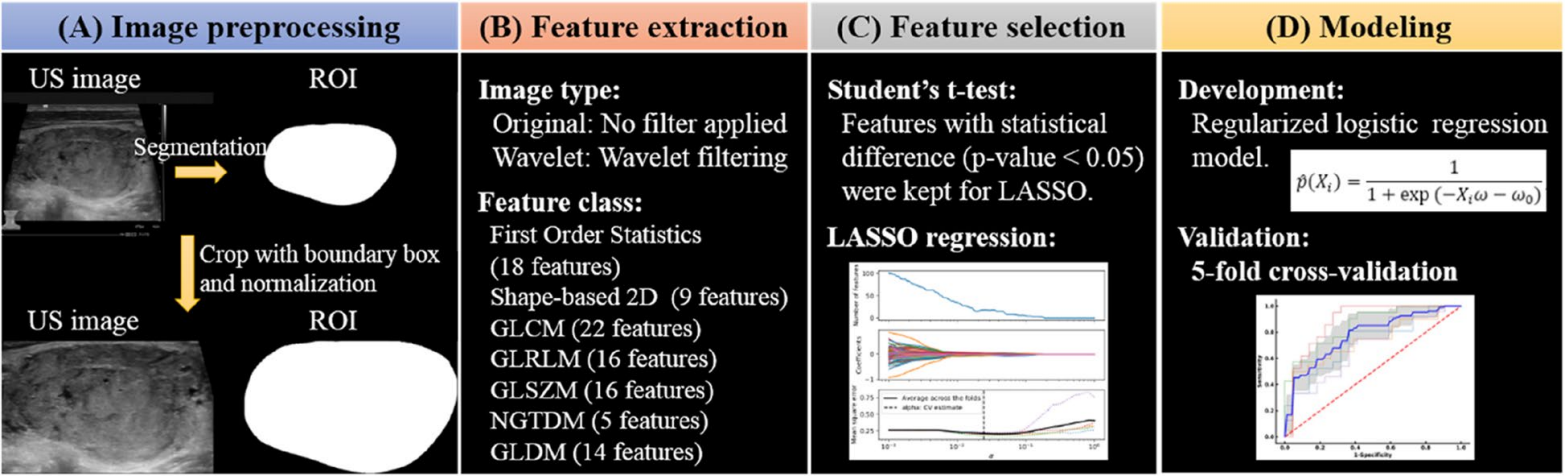
Workflow of a radiomics approach for prediction of lateral cervical lymph node metastases (LCLNM) in medullary thyroid carcinoma from preoperative thyroid ultrasound. **A** Preprocessing of thyroid ultrasound images included manual segmentation, cropping and normalization. **B** Radiomics features were extracted from preprocessed thyroid ultrasound images. **C** A two-step method of Student’s t-test and least absolute shrinkage and selection operator (LASSO) regression were used to select features for modeling. **D** A regularized logistic regression model was developed and validated in 5-fold cross-validation to predict the LCLNM status

Hence, the pixel intensity should range from 0 to 255. Figure 2(A) shows the ultrasound image preprocessing.

### Feature extraction, feature selection and modeling

#### Radiomics feature extraction and selection

Figure 2(B) and (C) present the process of feature extraction and feature selection. We extracted a total of 464 radiomics features using an open-source python package for the extraction of radiomics features from medical imaging (PyRadiomics) [23]. These features derived from original image and wavelet image (applying either a high or a low pass filter in each of the two dimensions to the original image and yields 4 decompositions per level) including the first order statistics (18x5 features), shape-based two dimension (9x1 features), gray level co-occurence matrix (GLCM, 22x5 features), gray level run length matrix (GLRLM, 16x5 features), gray level size zone matrix (GLSZM, 16x5 features), neighbouring gray tone difference matrix (NGTDM, 5x5 features), and gray level dependence matrix (GLDM, 14x5 features).

A two-step feature selection strategy was used to reduce the dimensions of the radiomics features. Considering that the radiomics features are continuous variables with a normal distribution, a Student's t-test was first employed to identify features strongly associated with LCLNM status. The Student's t-test is a parametric test that is used to determine if variables in two independent groups have the same distribution. Statistical significance was defined as *p*-value < 0.05. A total of 238 features that showed a statistically significant difference were retained for the subsequent feature selection. Next, we further selected radiomics features using the least absolute shrinkage and selection operator (LASSO) approach, which is a penalized variable selection method appropriate for the regression of high-dimensional data. Since we used 5-fold cross-validation, the radiomics features we selected varied from fold-to-fold. We select radiomics features with a probability of occurrence greater than 60% in 5-fold training dataset. The details of LASSO approach for selecting features are presented in Supplementary Material 1 (including Supplementary Table 1 and Supplementary Figures 1-10).

#### Model development and validation

Figure 2(D) shows the modeling process. Given the small sample and lack of external validation data, we utilized 5-fold cross-validation to split our data into training and test datasets to reduce the bias and risk of overfitting. Namely, the MTC patients were randomly divided into five equal parts, and the regularized logistic regression model was trained only on four parts (training dataset) and tested on the remaining part (test dataset). The probability of the LCLNM positive class $P\left({y}_{i}=1|{X}_{i}\right)$ is as following:

2 $$\widehat{p}\left({X}_{i}\right)=\frac{1}{1+{\text{exp}}(-{X}_{i}\omega -{\omega }_{0})}$$

where ${y}_{i}$ takes values in the set {0, 1} for the $i$ th patient, ${X}_{i}$ is features, and $\omega$ and ${\omega }_{0}$ are feature coefficients and intercept. The optimization process is to minimize a cost function:

3 $$min\,C\sum_{i=1}^{n}(-{y}_{i}{\text{log}}\left(\widehat{p}\left({X}_{i}\right)\right)-(1-{y}_{i}){\text{log}}(1-\widehat{p}\left({X}_{i}\right)))+r(\omega )$$

where $r(\omega )$ is the regularization penalty term and there are four choices as presented in Supplementary Table 2, and $C$ is the factor to adjust the inverse of regularization strength, and the regularization has the benefit of increasing stability.

For comparison, we established three models using the ultrasound features from TI-RADS, the radiomics features from preoperative thyroid ultrasound images, and the radiomics features combined with TI-RADS, respectively. The three models were separately denoted as TI-RADS model (TM), radiomics model (RM), and radiomics combined with TI-RADS model (RTM). The above features went through the feature selection and standard-scaled steps before being inputted into the models. In parallel, we computed a radiomics score for each patient based on a linear combination of the radiomics features weighted by their coefficients from RM when building RTM.

The hyperparameters for the model included optimizer, regularization penalty term ($r(\omega )$) and inverse of regularization strength ($C$). The optimization algorithm of ‘lbfgs’ was used as optimizer. The l_2 regularization term is applied as penalty. The inverse of regularization strength C is set to 1. The tolerance for stopping criteria is 1e-4. The maximum number of iterations taken for the solvers to converge is 1000.

As we adopted 5-fold cross-validation, the process of feature selection, model training, and testing steps is repeated five times so that each part is given a chance to be the independent test dataset. The analysis described above was implemented in Python software (version 3.8) with scikit-learn package (version 1.1.2).

### Model evaluation and statistical analysis

The performance of the model was evaluated by quantitative indexes including the area under the receiver operating characteristic (ROC) curve (AUC), accuracy (ACC), sensitivity (SEN), specificity (SPE), positive predictive value (PPV), negative predictive value (NPV), Matthew's correlation coefficient (MCC) and F1 score (F1), which are described in Supplementary Material 2.

MTCs with LCLNM negative and positive were compared based on patient demographic information. The Student’s t-test was used to determine whether there was any statistical difference in these features. A two-sided *p*-value < 0.05 was used as the criterion to indicate a statistically significant difference. Delong’s test was used to test whether there is a statistical difference in LCLNM status prediction for different models.

The decision curve analysis (DCA) was used to test the clinical usefulness of the regularized logistic regression model in LCLNM status prediction. The net benefit of the LCLNM positive group can be calculated as following:

4 $$Net\,benifit\,treated= \frac{TP}{n}-\frac{FP}{n}\frac{{P}_{t}}{1-{P}_{t}}$$

where $TP$ is the number of LCLNM positive patients correctly identified as LCLNM positive, $FP$ is the number of LCLNM positive patients identified as LCLNM negative, $n$ is the total number of patients, ${P}_{t}$ is the probability threshold. The details can refer to Supplementary Material 3. The analysis described above was also implemented in Python software (version 3.8).

## Results

### Patient characteristics

We present in Table 1 the demographic information of MTC patients with LCLNM negative and positive. Of the enrolled 218 MTC patients (Fig. 1), there were 111 (percentage of 50.5%; median age:49, range:13-78; number of male&female:39&72) patients with LCLNM negative and 107 (percentage of 49.5%; median age:50, range:21-75; number of male&female:63&44 ) with LCLNM positive. There were no significant differences between MTC patients with LCLNM negative and positive in age, preoperative ultrasound features of composition, echogenicity, shape, and echogenic foci (all *p*>0.05). Of the preoperative ultrasound features, smooth or ill-defined margin were more frequent in MTC patients with LCLNM negative while extra-thyroidal extension margin was more frequent in MTC patients with LCLNM positive (*p*<0.001). TI-RADS level were significant differences between MTC patients in LCLNM negative group and positive group (*p*<0.05), and MTC patients in LCLNM positive had higher incidences of TI-RADS level (TR5) compared to MTC patients in LCLNM negative.

### Selected features and their importance

For TM, the ultrasound features of margin and TI-RADS level (*p*<0.05) was selected using the Student's t-test and used to establish the model. The coefficients and intercept of the TM in 5-fold cross-validation were summarized in Supplementary Table 3. The feature of margin is the most crucial component in TM.

**Fig. 3:**
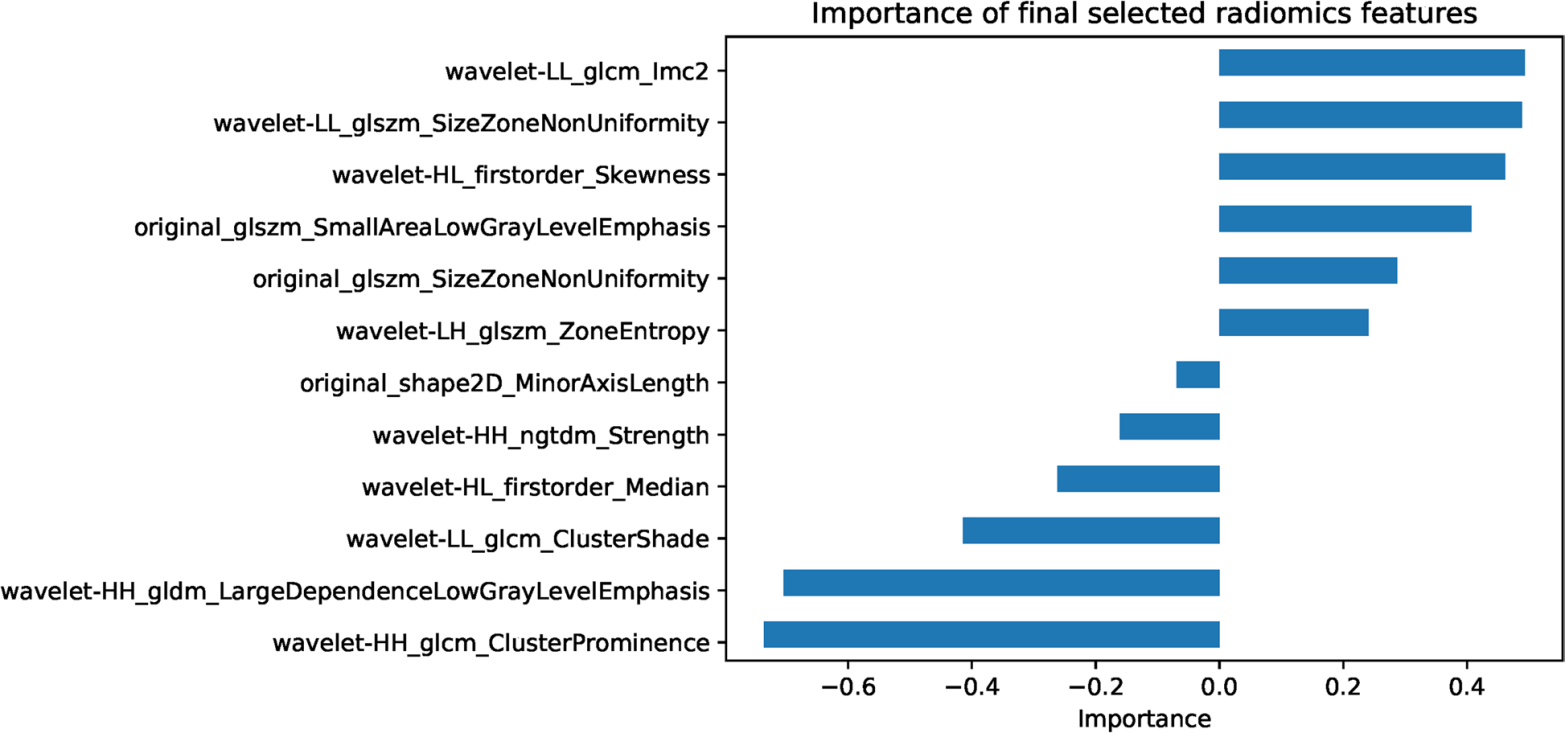
Importance of radiomics features for predicting lateral cervical lymph node metastases in medullary thyroid carcinoma

For RM, a total of 464 radiomics features from preoperative ultrasound imaging was first reduced to 238 (*p*<0.05) using the Student's t-test, and further reduced to 12 final selected radiomics features using the LASSO approach (the details can refer to Supplementary Material 1). Table 2 demonstrated the final selected radiomics features and their significant differences between LCLNM negative and LCLNM positive groups. We established the RM using the final selected radiomics features. The coefficients and intercept of the RM in 5-fold cross-validation were summarized in Supplementary Table 4. Since the coefficients of different features can reflect the feature importance, we summarized the selected radiomics feature importance (averaged coefficients of 5-fold cross-validation) as shown in Fig. 3. The feature is more important when having the larger absolute value

**Table 2:** Final selected radiomics features and the significant differences of the medullary thyroid carcinoma patients between lateral cervical lymph node metastases (LCLNM) negative and LCLNM positive. Data are presented as medians with ranges in parentheses

**Final radiomics features**	**LCLNM negative (*n*=111)**	**LCLNM positive (*n*=107)**	***p*-Value**
**First order statistics**			
wavelet-HL_firstorder_Median	4.2e-2 (-7.1e-2~0.3)	3.1e-2 (-8.5 e-2~0.1)	**<0.001**
wavelet-HL_firstorder_Skewness	-2.7e-2 (-14.3~14.4)	0.2 (-10.9~24.1)	**0.017**
**Shape-based features**			
original_shape2D_MinorAxisLength	145.9 (42.4~550.0)	220.3 (53.1~555.4)	**<0.001**
**GLCM**			
wavelet-LL_glcm_Imc2	0.98 (0.91~1)	0.98 (0.89~1)	**0.042**
wavelet-HH_glcm_ClusterProminence	0.5 (0.5~47.4)	0.6 (0.5~19.1)	**0.005**
wavelet-LL_glcm_ClusterShade	142.8 (-68.4~1303.6)	103.7 (-213.2~1018.4)	**0.029**
**GLSZM**			
original_glszm_SizeZoneNonUniformity	20.5 (1.9~122.5)	40.7 (3.6~440.9)	**<0.001**
wavelet-LH_glszm_ZoneEntropy	5.8 (4.3~6.6)	6.0 (4.4~6.6)	**<0.001**
original_glszm_SmallAreaLowGrayLevelEmphasis	2.8e-02 (0.3e-2~0.1)	3.6e-2 (0.6e-2~0.1)	**0.010**
waveletLL_glszm_SizeZoneNonUniformity	95.8 (9.8~533.5)	136.8 (9.0~1134.7)	**<0.001**
**NGTDM**			
wavelet-HH_ngtdm_Strength	7.5e-4 (3.1e-5~0.1)	2.1e-3 (1.9e-5~4.4e-2)	**0.001**
**GLDM**			
wavelet-HH_gldm_LargeDependenceLowGrayLevelEmphasis	4.6 (0.2~18.6)	2.2 (0.2~19.1)	**<0.001**

For RTM, we first computed a radiomics score and then combined with TI-RADS features to establish RTM. The coefficients of margin, TI-RADS Level and radiomics score and intercept of the model in 5-fold cross-validation were summarized in Supplementary Table 5. The radiomics score plays the most important role in RTM.

### Prediction performance of the models

**Fig. 4:**
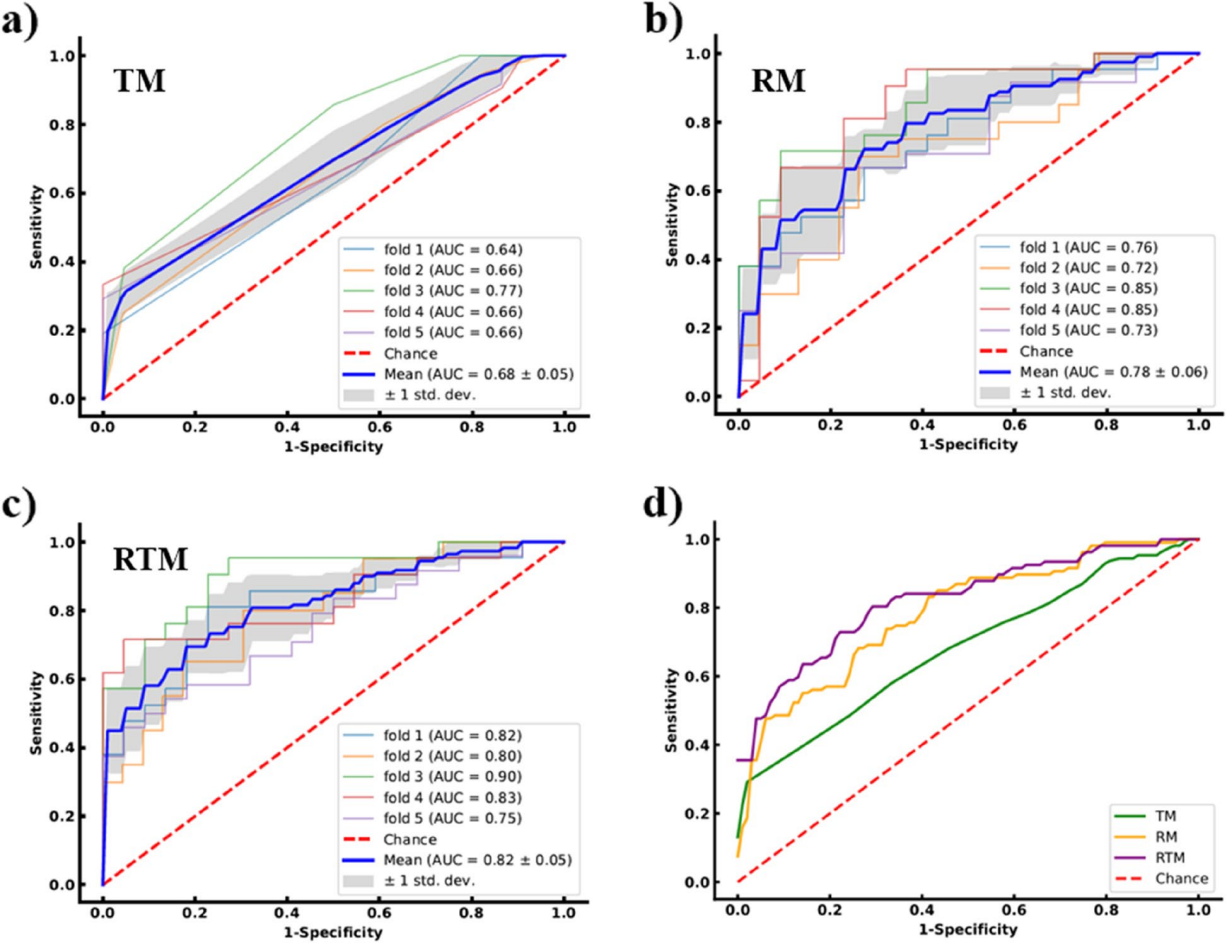
ROC curves of the LCLNM status prediction results of the three models in the 5-fold cross-validation independent test dataset. **a** ROC curves for TI-RADS model (TM), **b**) ROC curves for radiomics model (RM), **c**) ROC curves for radiomics combined with TI-RADS model (RTM), **d**) comparison of ROC curves by the three models. Shaded areas of **a**), **b**) and **c**) represent one standard deviation

Figure 4 illustrated the ROC curves of the LCLNM status prediction results of the three models in the 5-fold cross-validation independent test dataset. From the experimental results, the AUCs of the TM were 0.64, 0.66, 0.77, 0.66, and 0.66, respectively, and averaged to be 0.68±0.05 with one standard deviation. The AUCs of the RM were 0.76, 0.72, 0.85, 0.85, and 0.73, respectively, and averaged to be 0.78±0.06 with one standard deviation. The AUCs of the RTM were 0.82, 0.8, 0.9, 0.83, and 0.75, respectively, and averaged to be 0.82±0.05 with one standard deviation. The corresponding quantitative indexes of the three models and the Delong test results on all independent test dataset were summarized in Table 3.

**Table 3:** Quantitative indexes comparisons of TI-RADS model (TM), Radiomics model (RM) and radiomics combined with TI-RADS model (RTM) in 5-fold cross-validation independent test dataset

**Model**	**AUC**	**ACC**	**SEN**	**SPE**	**PPV**	**NPV**	**MCC**	**F1**
**TM**								
fold 1	0.64	0.56	0.67	0.45	0.54	0.59	0.12	0.6
fold 2	0.66	0.58	0.8	0.39	0.53	0.69	0.21	0.64
fold 3	0.77	0.67	0.86	0.5	0.62	0.79	0.38	0.72
fold 4	0.66	0.56	0.71	0.41	0.54	0.6	0.13	0.61
fold 5	0.66	0.57	0.75	0.36	0.56	0.57	0.12	0.64
Average	0.68	0.59	**0.76**	0.42	0.56	0.65	0.19	0.64
**RM**								
fold 1	0.76	0.67	0.62	0.73	0.68	0.67	0.35	0.65
fold 2	0.72	0.67	0.6	0.74	0.67	0.68	0.34	0.63
fold 3	0.85	0.7	0.76	0.64	0.67	0.74	0.4	0.71
fold 4	0.85	0.74	0.81	0.68	0.71	0.79	0.49	0.76
fold 5	0.73	0.65	0.67	0.64	0.67	0.64	0.3	0.67
Average	0.78	0.69	0.69	0.69	0.68	0.7	0.38	0.68
**RTM**								
fold 1	0.82	0.77	0.71	0.82	0.79	0.75	0.54	0.75
fold 2	0.8	0.7	0.65	0.74	0.68	0.71	0.39	0.66
fold 3	0.9	0.81	0.9	0.73	0.76	0.89	0.64	0.82
fold 4	0.83	0.79	0.71	0.86	0.83	0.76	0.59	0.77
fold 5	0.75	0.65	0.67	0.64	0.67	0.64	0.3	0.67
Average	**0.82**	**0.74**	0.73	**0.76**	**0.75**	**0.75**	**0.49**	**0.73**
**Significance level of Delong test results for different models**								
Test dataset	TM & RM			TM & RTM		RM & RTM		
All (5-fold)	0.030			<0.001		0.077		

The bold value represents the best value of a quantitative index

### Clinical utility

**Fig. 5:**
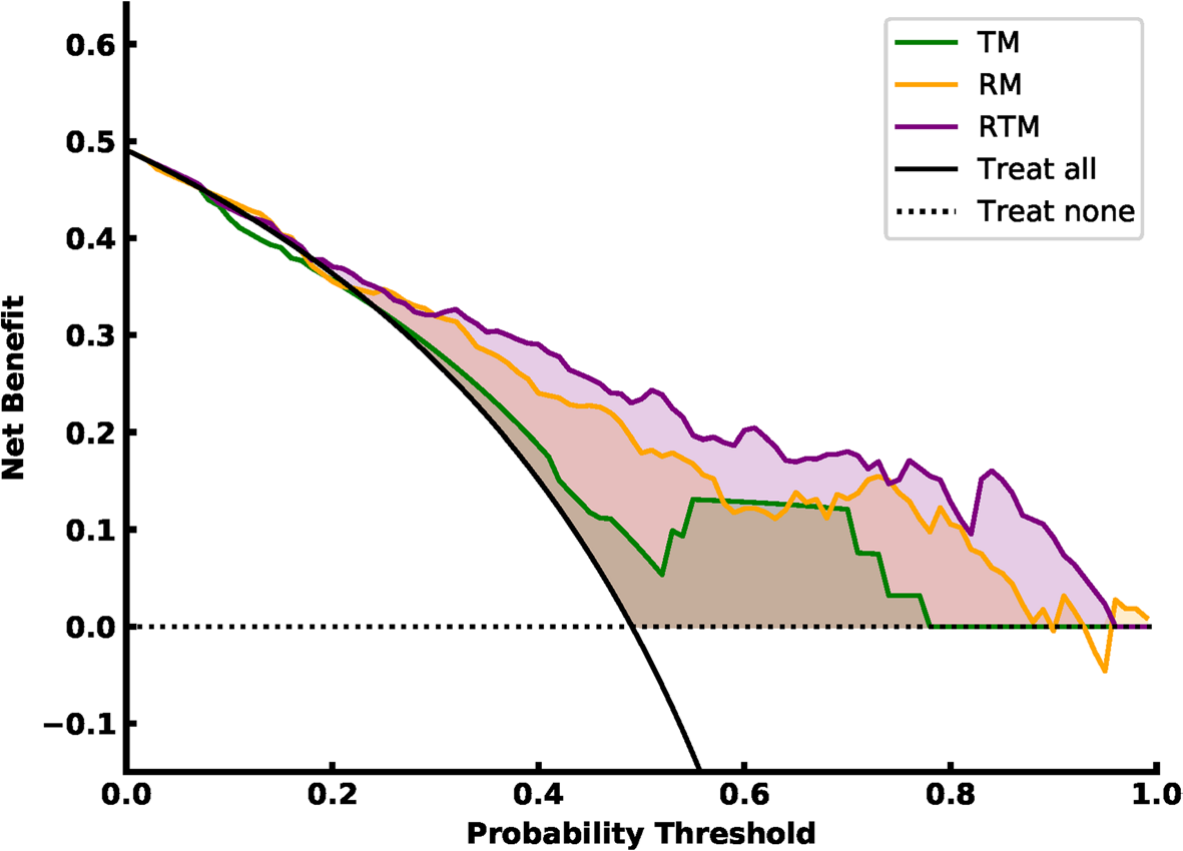
Comparison of decision curves of the LCLNM status prediction of the three models in all (5-fold) independent test dataset. (TM: TI-RADS model, RM: radiomics model, RTM: radiomics combined with TI-RADS model.)

Figure 5 illustrates the decision curve of the LCLNM status prediction models in all (5-fold) independent test dataset. The filled net benefit region demonstrated that using LCLNM status prediction models can gain more benefit than treating all MTC patients or treating no MTC patients. The RTM could bring more consistent and significant benefit to MTC patients than TM and RM.

## Discussion

Thyroid ultrasound is an effective tool to evaluate the lymph node status in MTC patients [6, 8]. We investigated the association of ultrasound features from TI-RADS and LCLNM status. There were significant differences between LCLNM negative group and LCLNM positive group in margin and TI-RADS levels, and these results are consistent with the previously reported results [9, 24]. Based on the two features, we build the regularized logistic regression model, that is TM, to predict LCLNM status. The feature importance analysis demonstrated that the margin was the more important predictor. Overall, the performance of TM as presented in Table 3 is not so good. Possible reasons may be that the TI-RADS results are commonly influenced by the experience of reviewers and more information from images have not been fully explored, as they are currently relying solely on the naked eye.

Many radiomics studies have investigated the capability for analyzing thyroid carcinoma, including prediction of distant metastases of follicular thyroid carcinoma [14], risk stratification systems for thyroid nodules [15], the differentiation between malignancy and benign thyroid nodules [25], prediction of lymph nodes status [18–21], prediction of BRAF mutation [26], prediction of malignancy and pathological outcome in patients with papillary thyroid cancer [27]. The relationship between radiomics and MTC, however, is not well understood. As far as we are aware, this study is the first to use radiomics to predict LCLNM in MTC patients. In this study, we extracted 464 radiomics features from ultrasound images and finally selected 12 features (Fig. 3) to establish the RM through a two-step feature selection approach. These features were distributed in first order statistics, GLCM, GLSZM, NGTDM and GLDM (Table 2). Some of these radiomics features, such as Small Area Low Gray Level Emphasis and Size-Zone Non-Uniformity, were similar to the previous study for prediction of distant metastasis of follicular thyroid carcinoma [14]. The Delong test results show the RM is significantly better than the TM (*p*<0.05) as shown in Table 3. Radiomics features can be taken as more powerful predictors.

Furthermore, we computed the radiomics score, referring to previous studies [14, 15], for each patient based on the selected 12 radiomics features. We established the RTM using the radiomics score, margin and TI-RADS levels. Compared with the RM, the performance of the RTM has been further improved as shown in Table 3, Figs. 3 and 4. The radiomics score played the most important role in the RTM. The Delong test results also show the RTM is significantly better than the TM (*p*<0.001). Overall, the RTM has the best performance for predicting LCLNM status in MTC patients.

In the absence of enough evidence of LCLNM, it is always questionable whether patients should undergo lymph node dissection. One of the main concerns with LCLNM is the risk of complications associated with extended lymphadenectomy, such as hypoparathyroidism and nerve palsy. Effective preoperative assessment for LCLNM status is essential. The performance of solely TI-RADS system to identify LCLNM in MTC patients is limited. Our model combining TI-RADS system and radiomics features have improved the prediction performance. It improved the ability to identify patients who require lymph node dissection while avoiding surgical complications for those who do not. This model can be used as an aid to clinical decision making when it is not clear to the clinician whether to perform lymph node dissection.

The stability and reproducibility of features are determined by ultrasound images. Ultrasound images can differ depending on the time and location they were taken, who performed the ultrasound with the probe, how much pressure was applied to the skin, patient status (e.g. body habitus, age, underlying medical conditions like skin diseases, or previous surgeries in the area), and other factors. Image normalization is a critical step that makes sure each pixel has a similar data distribution, allowing comparison with other images. Hence, the linear min-max normalization method was used to minimize this impact. On the other hand, we utilized 5-fold cross-validation to reduce the bias and maintain repeatability. Naturally, our current study lacks the validation of external data, and more data samples and involvement from more research centers will be part of our future research efforts in order to further enhance the stability of the model. Furthermore, there is often a gap between the statistical significance of radiomics features and their clinical relevance. Understanding and interpreting what these features represent in terms of underlying pathology can be challenging. This gap suggests that the practical utility of radiomics findings in clinical decision-making was only taken as a supporting tool, not as a decisive one. Additionally, the primary tumor is segmented manually, which limits the workflow's efficiency. The proposed model uses ultrasound information to help guide clinical decision-making solely from an ultrasound imaging perspective, and any other clinical information is not incorporated into the model. In the future study, a fully automated model would be developed that would include an automatic model for tumor segmentation and a radiomics-based and deep learning-based classifier comprising ultrasound and clinical information for LCLNM status prediction to further improve efficiency and accuracy.

## Conclusions

We proposed a radiomics-based model that can accurately predict the status of LCLNM in MTC patients, establishing the relationship between the radiomics and ultrasound features in MTC patients. This study is the first to use radiomics analysis to achieve accurate prediction of LCLNM using ultrasound information alone. This model has the potential to serve as an additional tool that helps determine the best course of action for treating this uncommon type of thyroid cancer.

### Supplementary Information

**Additional file 1:** **Supplementary Material 1.** LASSO feature selection. **Supplementary Material 2.** Quantitative Evaluation Indexes. **Supplementary Material 3.** Decision curve analysis (DCA). **Supplementary Figure 1.** The best  (equals to 0.02437) of LASSO feature selection for fold1. **Supplementary Figure 2.** LASSO selected features for fold1. **Supplementary Figure 3.** The best  (equals to 0.04398) of LASSO feature selection for fold2. **Supplementary Figure 4. **LASSO selected features for fold2. **Supplementary Figure 5. **The best  (equals to 0.03218) of LASSO feature selection for fold3. **Supplementary Figure 6.** LASSO selected features for fold3. **Supplementary Figure 7.** The best  (equals to 0.02899) of LASSO feature selection for fold4. **Supplementary Figure 8.** LASSO selected features for fold4. **Supplementary Figure 9.** The best  (equals to 0.02274) of LASSO feature selection for fold5. **Supplementary Figure 10.** LASSO selected features for fold5. **Supplementary Table 1.** Final selected radiomics features. **Supplementary Table 2.** Four choices for the regularization term  via the penalty. **Supplementary Table 3.** Coefficients and intercept of ACR TI-RADS model for LCLNM status prediction in 5-fold cross-validation. **Supplementary Table 4.** Coefficients and intercept of radiomics model for LCLNM status prediction in 5-fold cross-validation. **Supplementary Table 5.** Coefficients and intercept of radiomics combined with TI-RADS model for LCLNM status prediction in 5-fold cross-validation. Coefficients of w_1_, w_2_ and w_3_ are corresponding to Margin, TI-RADS Level and radiomics score.

## Data Availability

The datasets generated and/or analysed during the current study are not publicly available due to the hospital policy but are available from the corresponding author on reasonable request.

## Abbreviations

TI-RADS: Thyroid imaging reporting and data system
MTC: Medullary thyroid carcinoma
LCLNM: Lateral cervical lymph node metastases
ATA: American Thyroid Association
ACR: American College of Radiology
ROI: Region of interest
LASSO: Least absolute shrinkage and selection operator
PyRadiomics: Python package for the extraction of radiomics features from medical imaging
GLCM: Gray level cooccurence matrix
GLRLM: Gray level run length matrix
GLSZM: Gray level size zone matrix
NGTDM: Neighbouring gray tone difference matrix
GLDM: Gray level dependence matrix
TM: TI-RADS model
RM: Radiomics model
RTM: Radiomics combined with TI-RADS model
ROC: Receiver operating characteristic
AUC: Area under the ROC curve
ACC: Accuracy
SEN: Sensitivity
SPE: Specificity
PPV: Positive predictive value
NPV: Negative predictive value
MCC: Matthew's correlation coefficient
DCA: Decision curve analysis
