# Supplementary material for: Combining radiomics with thyroid imaging reporting and data system to predict lateral cervical lymph node metastases in medullary thyroid cancer
Source: BMC Med Imaging. 2024 Mar 18;24:64. doi: 10.1186/s12880-024-01222-7 (PMC10946103; doi:10.1186/s12880-024-01222-7)
Supplement: Supplementary file 1 — Additional file 1: Supplementary Material 1. LASSO feature selection. Supplementary Material 2. Quantitative Evaluation Indexes. Supplementary Material 3. Decision curve analysis (DCA). Supplementary Figure 1. The best (equals to 0.02437) of LASSO feature selection for fold1. Supplementary Figure 2. LASSO selected features for fold1. Supplementary Figure 3. The best (equals to 0.04398) of LASSO feature selection for fold2. Supplementary Figure 4. LASSO selected features for fold2. Supplementary Figure 5. The best (equals to 0.03218) of LASSO feature selection for fold3. Supplementary Figure 6. LASSO selected features for fold3. Supplementary Figure 7. The best (equals to 0.02899) of LASSO feature selection for fold4. Supplementary Figure 8. LASSO selected features for fold4. Supplementary Figure 9. The best (equals to 0.02274) of LASSO feature selection for fold5. Supplementary Figure 10. LASSO selected features for fold5. Supplementary Table 1. Final selected radiomics features. Supplementary Table 2. Four choices for the regularization term via the penalty. Supplementary Table 3. Coefficients and intercept of ACR TI-RADS model for LCLNM status prediction in 5-fold cross-validation. Supplementary Table 4. Coefficients and intercept of radiomics model for LCLNM status prediction in 5-fold cross-validation. Supplementary Table 5. Coefficients and intercept of radiomics combined with TI-RADS model for LCLNM status prediction in 5-fold cross-validation. Coefficients of w1, w2 and w3 are corresponding to Margin, TI-RADS Level and radiomics score. [file 12880_2024_1222_MOESM1_ESM.docx]

**Supplementary Material 1.** LASSO feature selection

To further select radiomics features, we used the least absolute shrinkage and selection operator (LASSO) method, which is a penalized technique for variable selection that is suitable for the regression of high-dimensional data. LASSO feature selection was performed using Python software (version 3.8) with scikit-learn package (version 1.1.2), where the function LassoCV (Lasso linear model with iterative fitting along a regularization path) was used to select features. The optimization objective for Lasso consists of a linear model with an added regularization term. The objective function to minimize is:

$$\min_{\omega} \frac{1}{2n_{samples}}{|\left| X\omega-y \right||}_{2}^{2}+\alpha{||\omega||}_{1}, (S1-1)$$

The lasso estimate thus solves the minimization of the least-squares penalty with $\alpha{||\omega||}_{1}$added, where $\alpha$ is a constant and ${||\omega||}_{1}$ is the $\mathcal{l}_{1}$-norm of the coefficient vector. We initially set $\alpha$ range from 1e^-3^ to 1 with 200 logspace. In the LassoCV function, cross-validation (CV) was used to select the best model (best $\alpha$) corresponding to the best selected features.

As we adopted 5-fold cross-validation to develop the classification model, the process of LASSO feature selection is repeated five times in different training dataset. Supplementary Figure 1 to Figure 10 show the best $\alpha$ and selected radiomics feature results from fold1 to fold5. We found the selected radiomics features varied from fold-to-fold. Hence, we select radiomics features with a probability of occurrence greater than 60% in 5-fold training dataset. In other words, the frequency of occurrence is at least 3 times and more in 5-fold training dataset as shown in Supplementary Table 1.

**Supplementary Material 2**. Quantitative Evaluation Indexes

The probability threshold was set to 0.5, and a predicted probability larger than 0.5 was classified as lateral cervical lymph node metastases (LCLNM) positive, and other values were classified as LCLNM negative. LCLNM positive was correctly identified as LCLNM positive, and this case was recorded as true positive (TP). LCLNM negative was identified as LCLNM positive, and this case was recorded as false negative (FN). LCLNM positive was identified as LCLNM negative, and this case was recorded as false positive (FP). LCLNM negative was correctly identified as LCLNM negative, and this case was recorded as true negative (TN). Then the accuracy (ACC), sensitivity (SEN), specificity (SPE), positive predictive value (PPV), negative predictive value (NPV), Matthew's correlation coefficient (MCC) and F1 score (F1) can be calculated as:

$$ACC=\frac{TP+TN}{TP+FP+TN+FN}, (s2-1)$$

$$SEN=\frac{TP}{TP+FN}, (s2-2)$$

$$ACC=\frac{TN}{TN+FP}, (s2-3)$$

$$PPV=\frac{TP}{TP+FP}, (s2-4)$$

$$NPV=\frac{TN}{TN+FN}, (s2-5)$$

$$MCC=\frac{TP\times TN-FP\times FN}{\sqrt{(TP+FP)(TP+FN)(TN+FP)(TN+FN)}}, (s2-6)$$

$$F1=2\times\frac{PPV\times SEN}{PPV+SEN}, (s2-7)$$

**Supplementary Material 3**. Decision curve analysis (DCA)

The decision curve analysis (DCA) was used to test the clinical usefulness of the regularized logistic regression model in LCLNM status prediction. The net benefit of the LCLNM positive group can be calculated as following:

$$Net benifit treated= \frac{TP}{n}-\frac{FP}{n}\frac{P_{t}}{1-P_{t}}, (s3-1)$$

$$Net benifit untreated= \frac{TN}{n}-\frac{FN}{n}\frac{P_{t}}{1-P_{t}}, (s3-2)$$

$$treated-treat all= untreated\frac{P_{t}}{1-P_{t}}, (s3-3)$$

$$Net benefit treat all=\frac{TP+FN}{n}-\frac{TN+FP}{n}\frac{P_{t}}{1-P_{t}}, (s3-4)$$

where $TP$ is the number of LCLNM positive patients correctly identified as LCLNM positive, $FN$ is the number of LCLNM negative patients identified as LCLNM positive, $FP$ is the number of LCLNM positive patients identified as LCLNM negative, $TN$ is the number of LCLNM negative patients correctly identified as LCLNM negative, $n$ is the total number of patients, $P_{t}$ is the probability threshold.


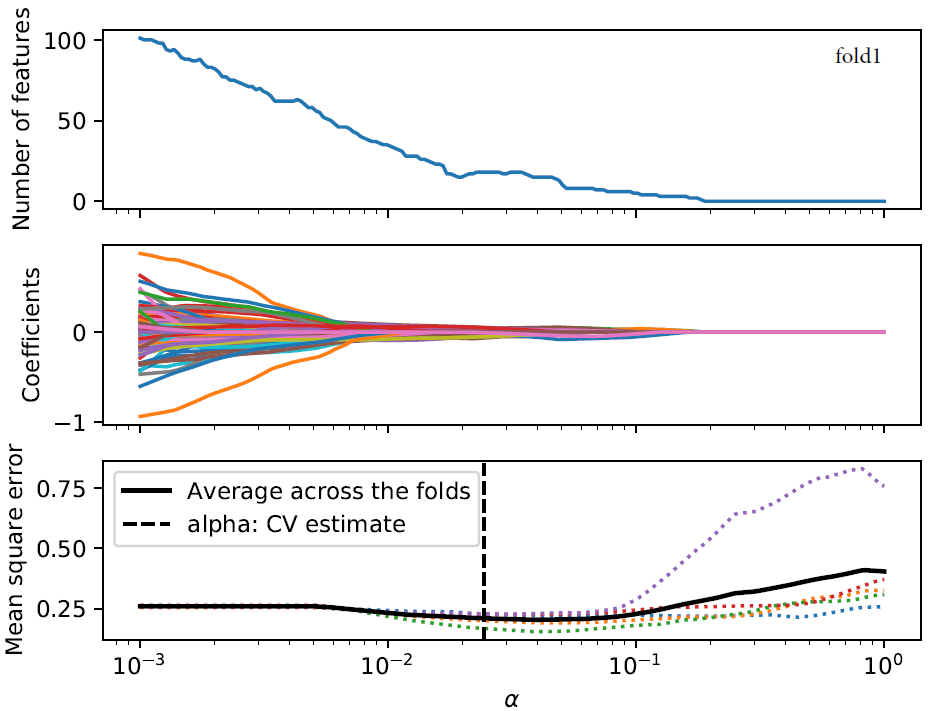
 **Supplementary Figure1.** The best $\alpha$ (equals to 0.02437) of LASSO feature selection for fold1.


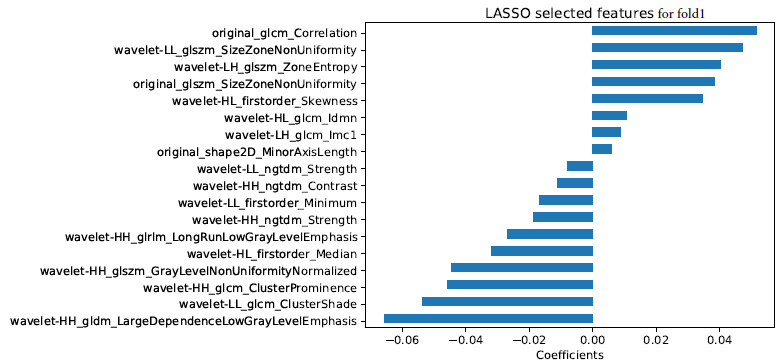


**Supplementary Figure2.** LASSO selected features for fold1.


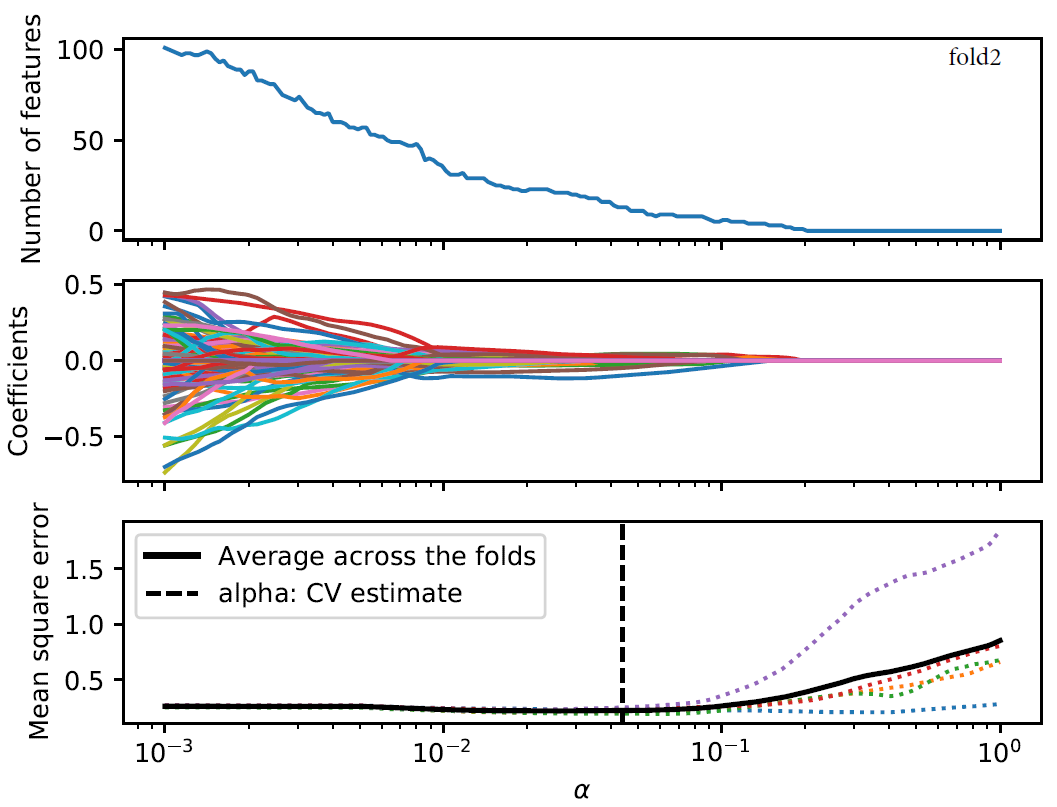


**Supplementary Figure3.** The best $\alpha$ (equals to 0.04398) of LASSO feature selection for fold2.


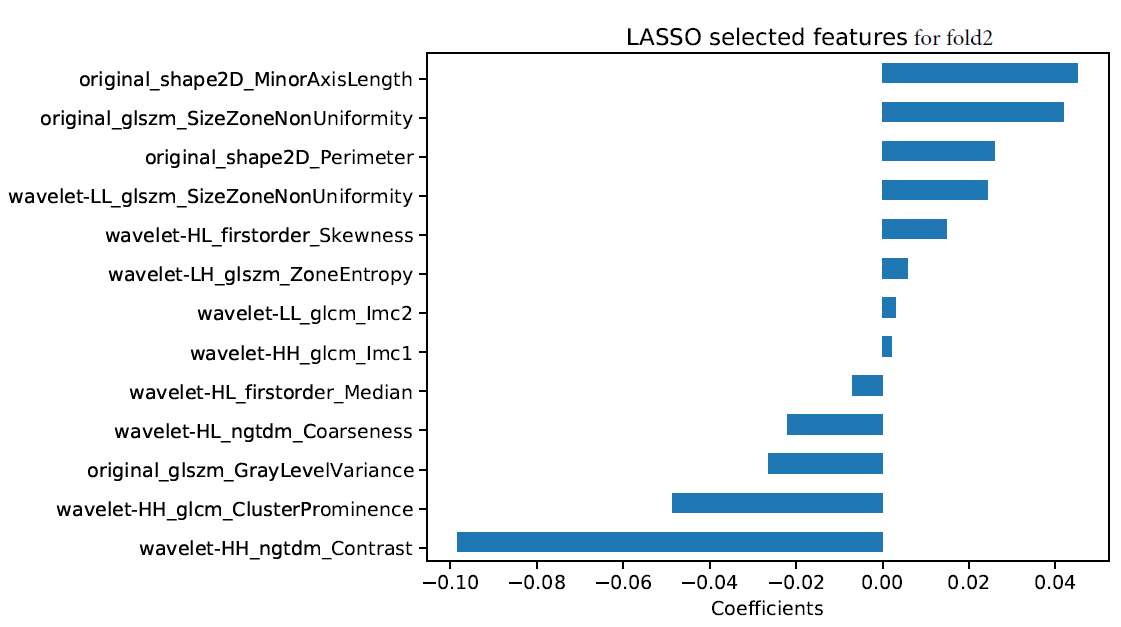


**Supplementary Figure4.** LASSO selected features for fold2.


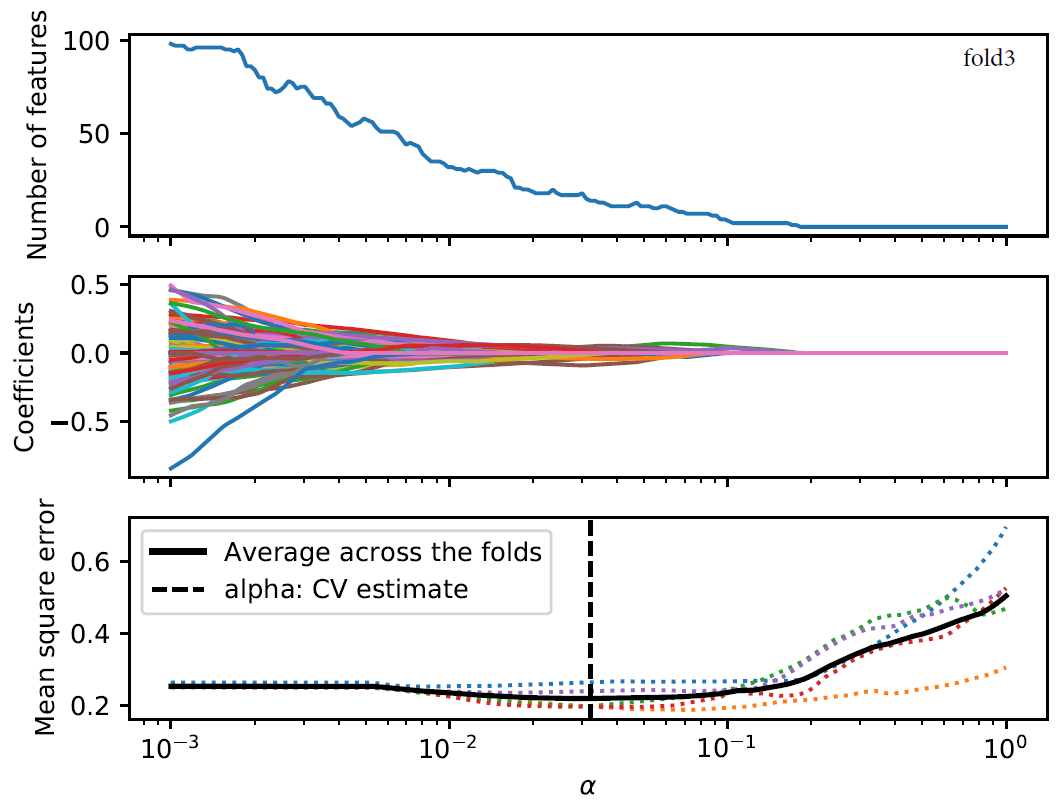


**Supplementary Figure5.** The best $\alpha$ (equals to 0.03218) of LASSO feature selection for fold3.


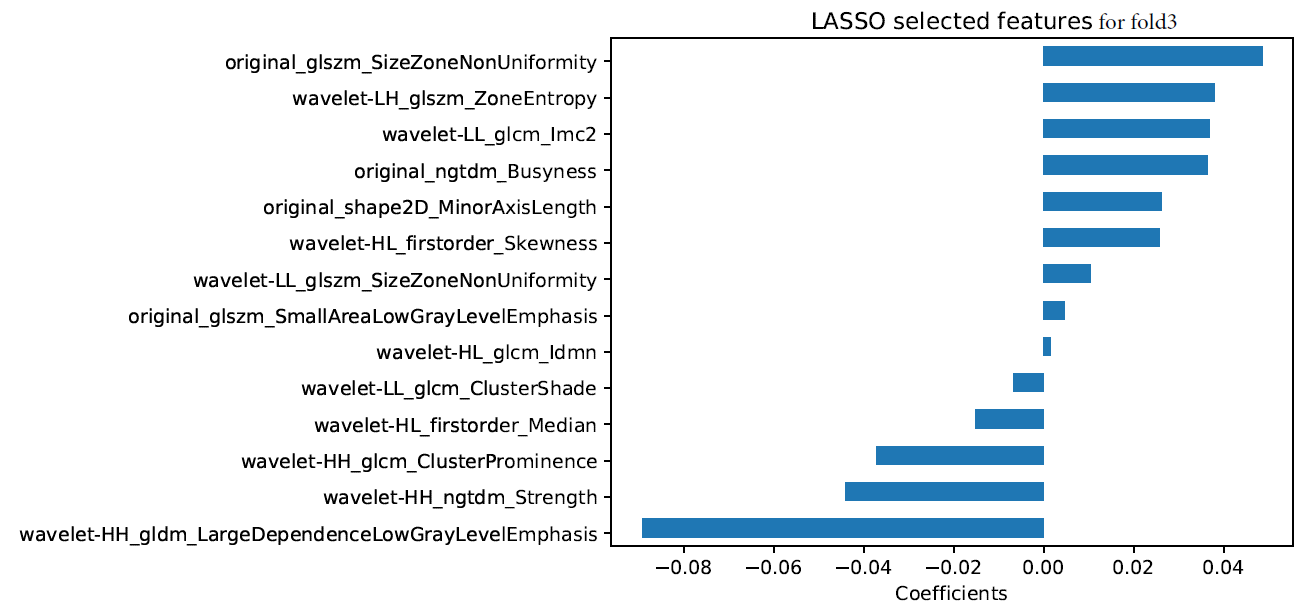


**Supplementary Figure6.** LASSO selected features for fold3.


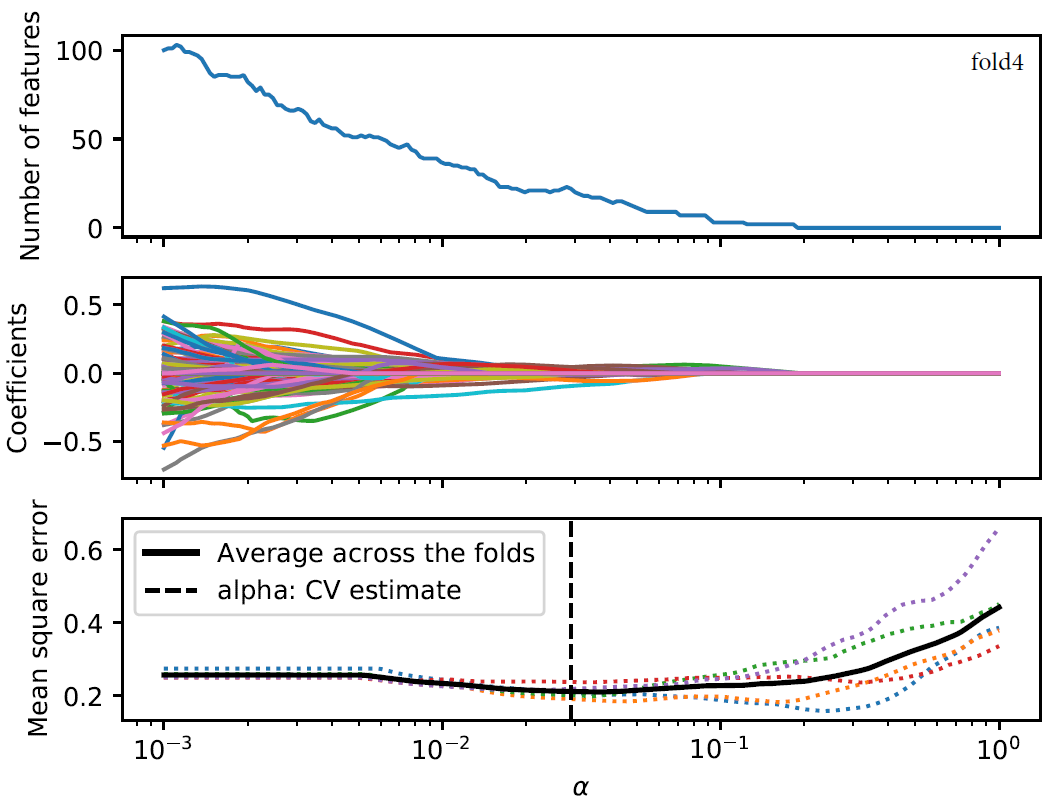


**Supplementary Figure7.** The best $\alpha$ (equals to 0.02899) of LASSO feature selection for fold4.


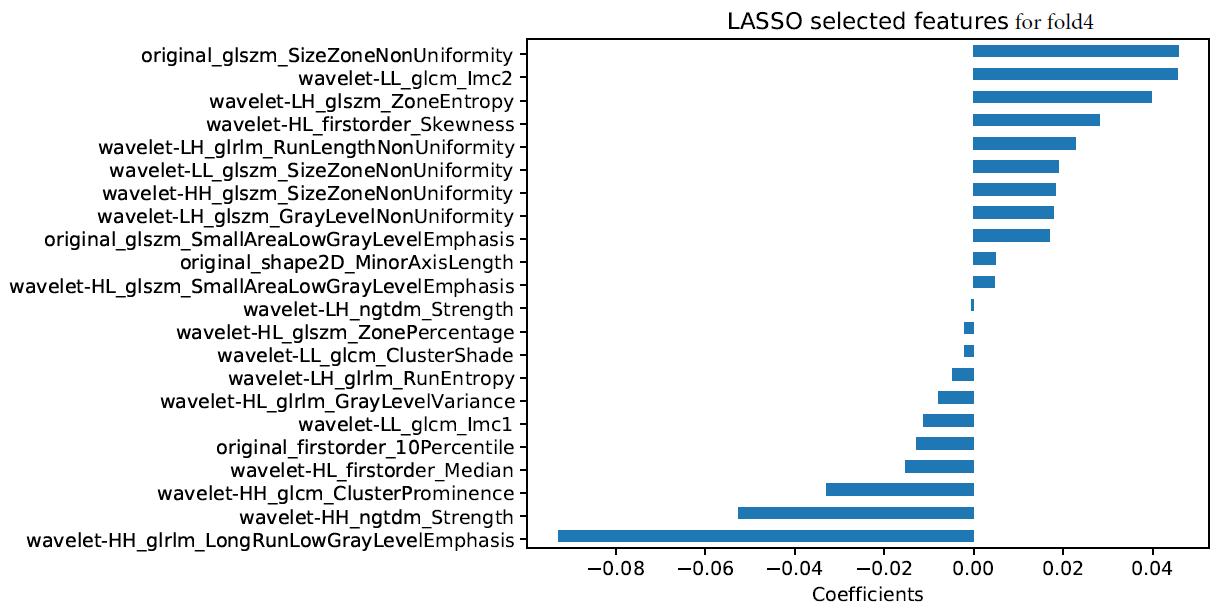


**Supplementary Figure8.** LASSO selected features for fold4.


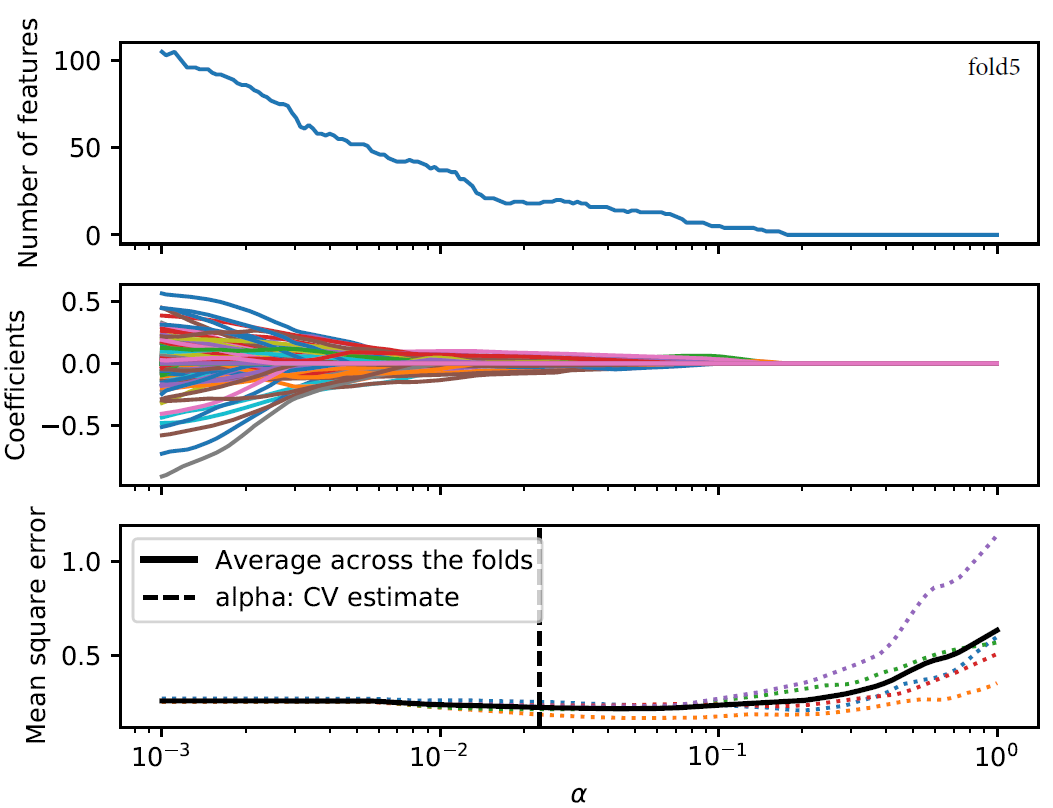


**Supplementary Figure9.** The best $\alpha$ (equals to 0.02274) of LASSO feature selection for fold5.


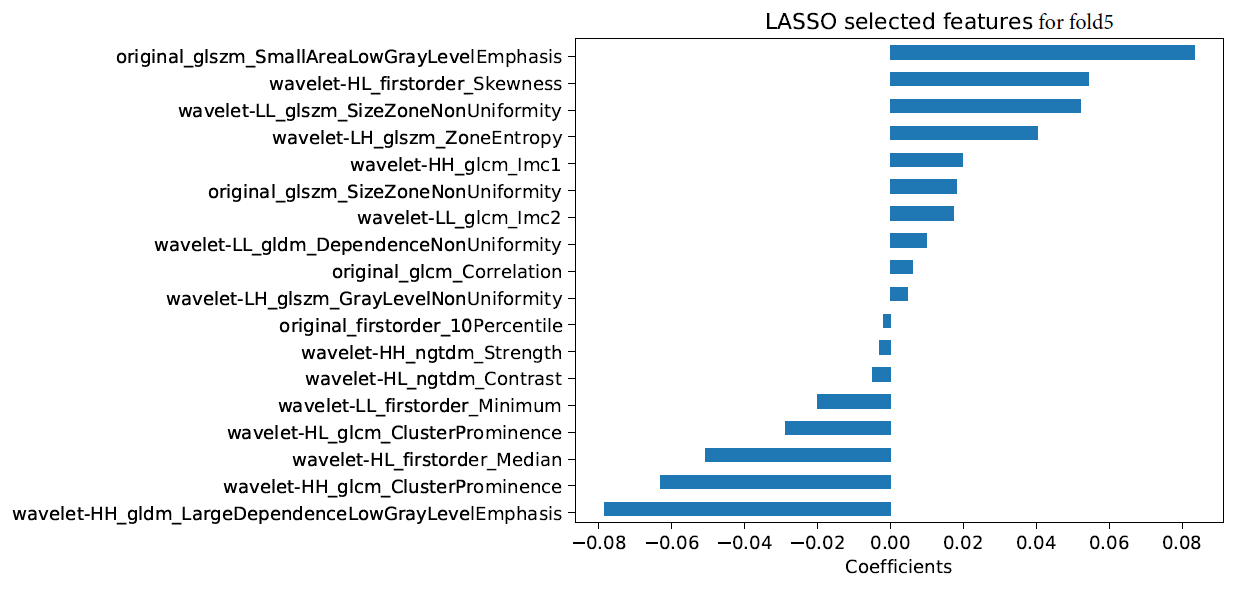


**Supplementary Figure10.** LASSO selected features for fold5.

**Supplementary Table 1**. Final selected radiomics features.

| Final selected radiomics features | frequency |
| --- | --- |
| original_shape2D_MinorAxisLength | 4 |
| original_glszm_SizeZoneNonUniformity | 5 |
| wavelet-LH_glszm_ZoneEntropy | 5 |
| original_glszm_SmallAreaLowGrayLevelEmphasis | 3 |
| wavelet-HL_firstorder_Median | 5 |
| wavelet-HL_firstorder_Skewness | 5 |
| wavelet-LL_glcm_Imc2 | 4 |
| wavelet-HH_glcm_ClusterProminence | 5 |
| wavelet-HH_ngtdm_Strength | 4 |
| wavelet-HH_gldm_LargeDependenceLowGrayLevelEmphasis | 3 |
| wavelet-LL_glcm_ClusterShade | 3 |
| wavelet-LL_glszm_SizeZoneNonUniformity | 5 |

**Supplementary Table 2**. Four choices for the regularization term $r(\omega)$ via the penalty.

| **penalty** | $\boldsymbol{r(\omega)}$ |
| --- | --- |
| $\mathcal{l}_{1}$ | ${\parallel\omega\parallel}_{1}$ |
| $\mathcal{l}_{2}$ | $\frac{1}{2}\omega^{T}\omega$ |
| ElasticNet* | $\frac{1-\rho}{2}\omega^{T}\omega+\rho{\parallel\omega\parallel}_{1}$ |
| None | 0 |

**Notes.** For ElasticNet*****, $\rho$ ($0\leq\rho\leq1$) controls the strength of $\mathcal{l}_{1}$ regularization versus $\mathcal{l}_{2}$ regularization. ElasticNet is equivalent to $\mathcal{l}_{1}$ when $\rho=1$ and equivalent to $\mathcal{l}_{2}$ when $\rho=$0.

**Supplementary Table 3**. Coefficients and intercept of ACR TI-RADS model for LCLNM status prediction in 5-fold cross-validation.

|  | **fold 1** | **fold 2** | **fold 3** | **fold 4** | **fold 5** |
| --- | --- | --- | --- | --- | --- |
| **ω_1_** | 0.7128 | 0.8544 | 0.7349 | 1.1452 | 0.9151 |
| **ω_2_** | 0.5177 | 0.3378 | 0.2651 | 0.3483 | 0.3605 |
| **ω_0_** | -3.8496 | -3.2369 | -2.6760 | -3.9450 | -3.5300 |

**Notes.** Coefficients of **ω_1_** and **ω_2_** are corresponding to Margin and TI-RADS Level features. **ω_0_** is the intercept.

**Supplementary Table 4**. Coefficients and intercept of radiomics model for LCLNM status prediction in 5-fold cross-validation.

|  | **fold 1** | **fold 2** | **fold 3** | **fold 4** | **fold 5** |
| --- | --- | --- | --- | --- | --- |
| **ω_1_** | -0.0017 | -0.0002 | 0.0000 | 0.0002 | -0.0015 |
| **ω_2_** | 0.0061 | 0.0052 | 0.0015 | 0.0050 | 0.0039 |
| **ω_3_** | 0.5852 | 0.4528 | 0.5686 | 0.5539 | 0.5543 |
| **ω_4_** | 7.6605 | 14.5757 | 21.1649 | 18.4566 | 30.3853 |
| **ω_5_** | -5.7642 | -6.2472 | -4.9997 | -3.9670 | -7.6741 |
| **ω_6_** | 0.1135 | 0.1263 | 0.1168 | 0.0949 | 0.1137 |
| **ω_7_** | 29.2647 | 26.4533 | 40.9056 | 38.1263 | 25.8843 |
| **ω_8_** | -0.0927 | -0.1294 | -0.1037 | -0.0788 | -0.1276 |
| **ω_9_** | -12.1214 | -6.0524 | -5.9156 | -12.4202 | -9.1387 |
| **ω_10_** | -0.1232 | -0.1147 | -0.0966 | -0.0986 | -0.0806 |
| **ω_11_** | -0.0042 | -0.0021 | -0.0022 | -0.0020 | -0.0017 |
| **ω_12_** | 0.0031 | 0.0038 | 0.0032 | 0.0026 | 0.0027 |
| **ω_0_** | -30.8461 | -28.2228 | -43.2354 | -40.4773 | -28.6071 |

**Notes.** Coefficients from **ω_1_** to **ω_12_** are corresponding to final selected radiomics features in Supplementary Material 1 Table 1. **ω_0_** is the intercept.

**Supplementary Table 5**. Coefficients and intercept of radiomics combined with TI-RADS model for LCLNM status prediction in 5-fold cross-validation. Coefficients of **ω_1_**, **ω_2_** and **ω_3_** are corresponding to Margin, TI-RADS Level and radiomics score.

|  | **fold 1** | **fold 2** | **fold 3** | **fold 4** | **fold 5** |
| --- | --- | --- | --- | --- | --- |
| **ω_1_** | 0.7795 | 0.8812 | 0.8599 | 1.0999 | 1.0281 |
| **ω_2_** | 0.5340 | 0.4116 | 0.3220 | 0.3172 | 0.4220 |
| **ω_3_** | 4.4318 | 4.4009 | 4.1755 | 3.7198 | 4.5265 |
| **ω_0_** | -6.2188 | -5.7850 | -5.1403 | -5.4582 | -6.1762 |

**Notes.** Based on a linear combination of the selected radiomics features weighted by their coefficients from the regularized logistic regression model as shown in Supplementary Table 3, we computed a radiomics score for each patient.
